# Adherence to the Mediterranean diet is associated with a higher BMD in middle-aged and elderly Chinese

**DOI:** 10.1038/srep25662

**Published:** 2016-05-09

**Authors:** Geng-dong Chen, Xiao-wei Dong, Ying-Ying Zhu, Hui-yuan Tian, Juan He, Yu-ming Chen

**Affiliations:** 1 Department of Medical Statistics & Epidemiology and Guangdong Provincial Key Laboratory of Food, Nutrition, and Health, School of Public Health, Sun Yat-sen University, Guangzhou, China

## Abstract

Previous studies showed that better adherence to the Mediterranean diet (MD) is associated with lower risk of chronic diseases, but limited data are available on bone health. We investigated the association of the MD with bone mineral density (BMD) in Chinese adults. We included 2371 participants aged 40–75 years in this community-based cross-sectional study. Dietary information was assessed at baseline and a 3-year follow-up. Alternate Mediterranean diet (aMed) scores were calculated. BMD was determined at the second survey. After adjusting for potential covariates, higher aMed scores were positively and dose-dependently associated with BMD (all P-trends < 0.05). The BMD values were 1.94% (whole body), 3.01% (lumbar spine), 2.80% (total hip), 2.81% (femur neck), 2.62% (trochanter), and 2.85% (intertrochanter) higher in the quintile 5 (highest, vs. quintile 1) aMed scores for all of the subjects (all P-values < 0.05). Similar associations were found after stratifying by gender (P-interaction = 0.338–0.968). After excluding the five non-significant components of vegetables, legumes, fish, monounsaturated to saturated fat ratio, and alcohol intake from the aMed scores, the percentage mean differences were substantially increased by 69.1–150% between the extreme quintiles. In conclusion, increased adherence to the MD shows protective associations with BMD in Chinese adults.

Osteoporosis and relative fractures, characterized by low bone mass, present great economic and health challenges worldwide^1^. Increasing evidence has shown that nutritional factors may play an important role in the development and prevention of osteoporosis through its life-scope influence^2^. Epidemiology studies have suggested that various single foods or nutrients have protective (e.g., calcium and Vitamin D^3^, vegetables and fruits^4^) or detrimental (e.g., saturated fat^5^) effects on bone health. However, few studies have examined the associations of general dietary pattern(s) with bone health.

The Mediterranean diet (MD) is a habitual diet adhered to in Mediterranean countries (e.g., Greece, Italy). Many studies have shown that adherence to the MD is associated with a lower risk of many chronic diseases (e.g., coronary heart disease^6^, stroke^6^, cognitive disorders^7^ and some cancers^8^,^9^,^10^) in populations worldwide. These results have suggested that the MD may be beneficial in preventing a variety of chronic diseases. Although several studies have been done in Caucasians, while inconsistent results were found, and few studies have included Asians subjects. In the EPIC cohort study, Benetou *et al.* found that an increased adherence to the MD was associated with a 7% (95%CI: 2–11%) decrease in hip fracture incidence per 1-unit increase in the MD scores in 48,814 men and 139,981 women^11^. Similar association with calcareous bone mineral density (BMD) was observed in a cross-sectional study of 200 Spanish women^12^. However, the MD scores were found to have null associations with bone fractures in an 8-year prospective study of 1,482 French elders^13^ and with lumbar spine BMD in a cross-sectional study of 196 Greek women (48 ± 12 years)^14^. Data from other populations (e.g., Asian populations) with different habitual diet patterns are scarce in this field. Our groups recently reported a beneficial association between a high alternate Mediterranean diet (aMed) score and lower hip fracture risk in a case-control study^15^. However, whether the beneficial association between adherence to the MD and fracture risk is caused by a pre-protection of BMD remains unclear due to the small sample sizes of the aforementioned former studies. Moreover, the aMed scale was developed for general health, and it remains uncertain whether it can be improved for the assessment of bone health. Therefore, studies that focus on BMD are valuable and urgently required.

The purpose of this cross-sectional study was to investigate the association of the MD (assessed via aMed scores) with BMD at the whole body, lumbar spine, and hip sites in middle-aged and elderly Chinese.

## Methods

### Study Participants

**Figure 1:**
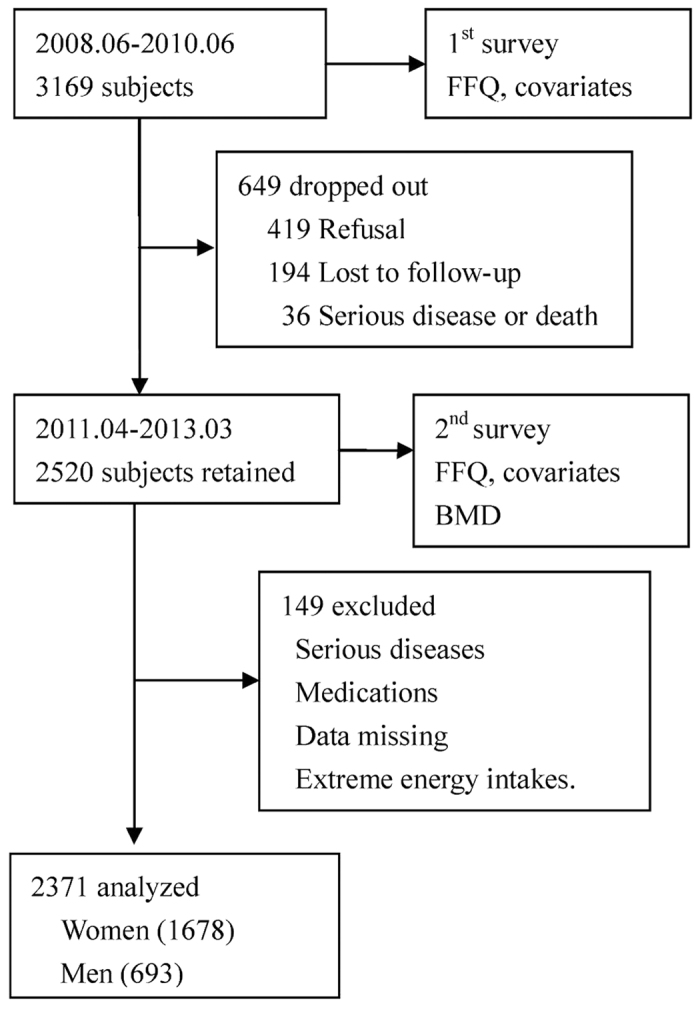
Flow chart of study participants.

The study was based on the Guangzhou Nutrition and Health Study (GNHS), a community-based prospective cohort study designed to investigate the nutritional determinants of cardiometabolic outcomes and osteoporosis. We recruited 3,169 subjects aged 40–75 years who had lived in urban Guangzhou for more than 5 years via advertisements and subject referrals between June 2008 and June 2010. After about three years between April 2011 and March 2013, 2,520 subjects of them were followed up, while 649 subjects dropped out due to refusal (419 subjects), loss of contact or emigration (194 subjects), or serious disease or death (36 subjects). A questionnaire survey was conducted to collect habitual dietary intake and various covariates at both baseline and follow-up, and BMD was measured at follow-up only. We further excluded 149 subjects for the following reasons: (i) history of serious disease, such as malignancy or hyperthyroidism; (ii) history of medications for osteoporosis; (iii) missing core data; and (iv) extreme energy intakes (<800 or >4,200 kcal/d for men and <600 or >3,500 kcal/d for women). In the end, 2,371 subjects (containing 1,678 women and 693 men) who completed the two surveies and BMD measurements were included in the cross-sectional study (Fig. 1). All of the subjects provided written informed consent. This study was performed in accordance with relevant guidelines and regulations by the Ethics Committee of the School of Public Health at Sun Yat-sen University.

### Measurements and Data Collection

Subjects were invited to the School of Public Health at Sun Yat-sen University to provide relevant measurements and engage in face-to-face interviews at baseline and follow-up. Structured questionnaires were used to collect information related to demographics (e.g., age, gender, education, martial status, household income); habitual dietary intake; other lifestyle factors (e.g., smoking status, alcohol drinking, physical activities); and history of diseases, medications, and use of supplements (e.g., multivitamin use, calcium supplements use, oral estrogen). Current smokers were defined as those who smoked at least one cigarette per day for the last 6 months. Physical activity was measured and translated into MET·h/d as described previously^16^. The subjects’ heights and weights were measured with the subjects in a standing position wearing light clothing and no shoes. Their body mass indexes (BMIs, in kg/m^2^) were then calculated.

### Assessment of Dietary Intake

A pre-validated 79-item food-frequency questionnaire (FFQ)^17^ was used to collect the subjects’ dietary information. The subjects were asked to report the frequencies (never, per year, per month, per week, and per day) and approximate portion sizes of the foods they consumed during the preceding year based on provided pictures. The average daily intake of total energy and specific nutrients were then calculated according to the China Food Composition Table 2002^18^. Average values of the dietary data collected at baseline and follow-up were used for the calculation of the MD scores in the 2,371 subjects.

### Alternate Mediterranean Diet (aMed) Score

Adapted from the MD score^19^ used by Fung *et al.*^6^, the aMed score reflects an adaptation of the principles of the traditional MD to non-Mediterranean countries. In this study, the score was calculated based on a scale including nine components: whole grains, vegetables (excluding potatoes), fruits (including juices), legumes, nuts, fish, ratio of monounsaturated fat (MUF) to saturated fat (SF), red and processed meats, and alcohol. All nine of these components were adjusted for the total energy intake using the residual method^20^. One (or zero) point was assigned to each component and the aMed score was calculated as described previously^15^. The total aMed score ranged from 0 to 9, and subjects with higher scores were considered to have adhered to a diet more resembling the MD. The Spearman correlation coefficient between the aMed scores at baseline and follow-up of the 2,371 followed subjects was 0.333 (P < 0.001).

### BMD Assessment

BMD (g/cm^2^) at the whole body (WB), lumbar spine (LS), total hip (TH), femur neck (FN), trochanter (TR), intertrochanter (IN) and Ward’s triangle (WT) area sites was measured using dual-energy X-ray absorptiometry (DXA) (Discovery W, Hologic Inc., Waltham, MA, USA) and analyzed with Hologic Discovery software version 3.2 during the follow-up (April 2011 and March 2013). The *in-vivo* coefficients of variation of the duplicated BMD measurements in 30 subjects after repositioning were 1.18% (WB), 0.87% (LS), 1.02% (TH), 1.92% (FN), 1.82% (TR), and 2.35% (IN), respectively. The long-term CV of the measurements was 0.26%, a value found by testing the phantom daily between March 2011 and May 2015.

### Statistical Analysis

Common characteristics were presented as means and standard deviations (SDs) for the continuous variables and as frequencies and percentages for the categorical variables.

The aMed scores were calculated by adding the point values assigned to each food group according to the gender-median intake cutoffs. Men and women had similarly distributed aMed scores and both were grouped into quintiles 1 (lowest) to 5 (highest) based on the points they received, i.e., 0–2, 3, 4, 5, and 6–9, respectively. We used multivariate analyses of covariance to compare the covariate-adjusted BMD means of the quintiles by aMed score. Two covariance models were used with Model I adjusted for age and sex, and Model II further adjusted for BMI, marital status, education, household income, smoking status, calcium supplement use, multivitamin use, physical activity, and daily total energy intake. Stratified analyses were performed according to gender, and years since menopause and use of estrogen were added as factors for females only. Bonferroni tests were conducted to make multiple comparisons between quintiles. A two-sided P-value < 0.05 was considered statistically significant. All of the analyses were performed with SPSS 17.0 for Windows (SPSS, Inc., Chicago, USA).

## Results

Our study included 1,678 women and 693 men (Table 1). The mean (SD) age was 59.5 (4.7) years for women (96.8% of whom were postmenopausal) and 62.1 (5.2) years for men. As the aMed scores increased from quintiles 1 to 5 (highest), the subjects tended to have higher household incomes; be more educated; have higher dietary intakes of protein, carbohydrate, whole grain, vegetables, fruits, legumes, nuts, fish, MUF/SF, but lower intakes of total fat, saturated fat, MUF, and red and processed meats; be older; be married; be more likely to use multivitamin supplements; engage in more vigorous physical activity; and smoke less (all P-values < 0.05).

Higher aMed scores were positively and dose-dependently associated with higher BMDs at all of the bone sites (2.41–3.96% higher, quintile 5 vs. quintile 1, all P-values < 0.001), except Ward’s triangle area, after adjusting for age and gender in the subjects (Table 2). Similar associations were retained but slightly attenuated by other variables (e.g., BMI, education, smoking, physical activity, etc.) further adjusted in Model II. The BMD values were 1.94% (WB), 3.01% (LS), 2.80% (TH), 2.81% (FN), 2.62% (TR),and 2.85% (IN) higher in the top (vs. bottom) aMed score quintiles for all of the subjects (all P-values < 0.01). Similar associations were found in both women and men (P-interaction = 0.338–0.964) as shown in Table 3.

We also examined the associations between each aMed component and BMD. Of the nine components, higher intakes of whole grain, fruit, nuts, and a lower intake of red and processed meats were significantly associated with a higher BMD at several bone sites. No significant associations were found for the other five components (vegetable, legume, fish, MUF/SF, and alcohol) in this study (Supplemental Table 1). After excluding the non-significant components from the calculation of the aMed scores, more significant associations were observed. The mean difference percentages increased by 121% (WB), 117% (LS), 70.0% (TH), 93.6% (FN), 69.1% (TR), 82.5% (IN), and 150% (WT) between the extreme quintiles (Table 4).

## Discussion

A favorable association between adherence to the MD and BMD was observed in a large community-based cross-sectional study of middle-aged and elderly Chinese. Our findings suggested that the aMed scale is a useful index for assessing appropriate diet quality for BMD. The results highlighted the potential importance of adherence to the MD in improving bone health.

Although the MD has been associated with a lower risk of many chronic diseases in populations worldwide, its association with bone health and especially BMD is less well known. The favorable associations observed in our study were consistent with those in several other studies but not all studies. Increased adherence to the MD was associated with a 7% (95%CI: 0.02–0.11) decrease in hip fracture incidence per 1-unit increase in the MD scores in 48,814 men and 139,981 women (49 ± 11 years) at a 9-year follow-up in the EPIC study^11^. Our group recently found a similar favorable association between higher aMed scores and a lower risk of hip fracture (OR 0.28, 95%CI 0.18–0.43) in a case-control study of 726 pairs (case/control) of elderly Chinese subjects (55–80 years)^15^. A similar protective association with calcareous bone BMD (P-trend = 0.001) was also observed in a cross-sectional study of 200 Spanish women^12^. However, null associations were found with the risk of hip, vertebral, and waist fractures in 1,482 French elders (>65 years) in an 8-year prospective study^13^ and with lumbar spine BMD in another cross-sectional study of 196 Greek women (48 ± 12 years)^14^. The non-significant results of these two studies might have been caused by their smaller sample sizes, discrepancies in the different methods or indexes used to assess adherence to the MD, or diverse outcomes at the different sites. Data from Asian subjects in this field are scarce. Nevertheless, the favorable associations between the aMed scores and BMD and the risk of hip fracture found in this study and in our previous study of Chinese adults highlights the importance and potential practical value of the MD in improving BMD and preventing hip fractures in Chinese populations.

Of the nine individual components of the aMed score, four were associated with BMD in this study. Higher intakes of whole grains, fruits, and nuts and a lower intake of red and processed meat were independently associated with higher levels of BMD at several bone sites. Consistent with our results, many studies have associated sufficient intakes of plant foods and their phytonutrients and lower intakes of red and processed meat in diets with a higher BMD^12^,^21^,^22^,^23^,^24^. However, although they have shown beneficial associations in other studies, the other five components (vegetable, legume, fish, MUF/SF, and moderate alcohol consumption) showed no independent associations with BMD in this study^5^,^25^,^26^,^27^,^28^. The binary classification might attenuate the associations between these nutrients and BMD. Besides, the low intakes of legume and plant-based MUF and the “bottom-up” pattern (either never or excessive drinking) of alcohol drinking might have partly accounted for the null association in this population. Moreover, when we ruled out the five non-significant components from the original score, the favorable associations between the aMed score and BMD tended to be more significant. Our findings suggested that the aMed score may need to be improved to evaluate bone-specific diet quality among different populations. Additional longitudinal studies are required to clarify this issue.

The favorable associations between these components of the aMed score and BMD may result from their nutrients and other nutritional components. For example, calcium; potassium^29^; vitamins B^30^, C^31^, and K^32^; carotenoids^33^, and flavonoids^34^ rich in fruits; and Vitamin E rich in nuts^35^ showed beneficial associations with BMD as previously reported. In addition, the MD may prevent osteoporosis through an anti-inflammatory path. Better adherence to the MD was associated with lower levels of several pro-inflammatory cytokines (e.g., C-reactive protein and interleukin-6)^36^,^37^. Overproduction of these pro-inflammatory cytokines was associated with higher osteoclastic bone resorption rates and an increased risk of osteoporosis^38^. Moreover, the MD may provide oxidation resistance against oxidative stress and reactive oxygen species^39^, which inhibits the differentiation of osteoblastic cells and plays an important role in the development of osteoporosis^40^.

This study has several strengths. First, to our knowledge, it is the first study to examine the association between the MD (aMed scores) and BMD based on a large sample size. Second, the averages of dietary data were used for analyses in this study, providing a better estimation of the intake situation at follow-up. Finally, BMD was scanned at multiple sites, which enabled us to achieve a full-scale understanding of the association.

Our study has several limitations. First, the cross-sectional design could not infer a causal association, although we used average values of dietary intake to better estimate habitual consumption over the period before the BMD assessment, attenuating the possibility of causal inversion. Second, although we carefully adjusted for a variety of BMD-related confounders, residual cofounding might still nevertheless occurred in our study due to measurement errors and the limited number of covariates that could be measured. Finally, the subjects, who were recruited as volunteers, might have led healthy lifestyles or engaged in healthy activities. However, health-related factors, economic and education statuses, smoking, and the use of calcium supplements and multivitamins did not significantly modify the aMed-BMD association (P-interaction range: 0.051–0.877).

In conclusion, we found that better adherence to the MD (indicated by higher aMed scores) was favorably associated with BMD in middle-aged and elderly Chinese. The associations tended to be more significant when five non-significant components were excluded. The results suggest that bone-specific MD scores may be required to evaluate bone-related diet quality in this population. Large-scale and long-term prospective studies are required to better address these results.

## Additional Information

**How to cite this article**: Chen, G.-d. *et al.* Adherence to the Mediterranean diet is associated with a higher BMD in middle-aged and elderly Chinese. *Sci. Rep.* **6**, 25662; doi: 10.1038/srep25662 (2016).

## Supplementary Material

Supplemental Table 1

## Figures and Tables

**Table 1:** Characteristics of study participants by quintile of aMed score.

	Quintile of aMed score for total subjects^a^					P-trend
	Q1	Q2	Q3	>Q4	Q5(highest)	
**N** = **2371**	448	478	502	495	448	
**aMed Score (range)**	0–2	3	4	5	6–9	
Age, year	59.7 (4.84)	60.6 (4.89)	60.2 (4.86)	60.2 (4.86)	60.7 (5.56)	**0.036**
Body mass index, kg/m^2^	23.3 (3.29)	23.6 (3.15)	23.3 (2.98)	23.7 (3.14)	23.7 (3.27)	0.065
Household income, N (%)						**0.002**
<2000 Yuan·m^−1^ P^−1^	101 (22.5)	86(18.0)	84 (16.7)	83 (16.8)	75 (16.7)	
2000–3000 Yuan·m^−1^· P^−1^	188 (42.0)	202 (42.3)	209 (41.6)	193 (39.0)	176 (39.3)	
>3000 Yuan·m^−1^· P^−1^	159 (35.5)	190 (39.7)	209 (41.6)	219 (44.2)	197 (44.0)	
Education, y						**0.036**
<9	132 (29.5)	147 (30.8)	140 (27.9)	117 (23.6)	121 (27.0)	
9–12	227 (50.7)	215 (45.0)	233 (46.4)	253 (51.1)	218 (48.7)	
>12	89 (19.9)	116 (24.3)	129 (25.7)	125 (25.3)	109 (24.3)	
Married, N (%)	391 (87.3)	425 (88.9)	447 (89.0)	445 (89.9)	398 (88.8)	0.167
Smoker^b^, N (%)	47 (10.5)	44 (9.2)	46 (9.2)	39 (7.9)	30 (6.7)	**0.033**
Calcium supplement user, N (%)	116 (25.9)	132 (27.6)	162 (32.3)	150 (30.3)	139 (31.0)	0.055
Multivitamin regular use, N (%)	68 (15.2)	82 (17.2)	89 (17.7)	102 (20.6)	94 (21.0)	**0.008**
Physical activity^c^, MET• h/d	33.9 (5.50)	33.4 (4.78)	34.3 (5.70)	34.1 (5.85)	34.7 (6.08)	**0.007**
Dietary intake^d^						
Energy intake, kkcal/d	1.64 (0.41)	1.64 (0.38)	1.60 (0.39)	1.65 (0.39)	1.63 (0.40)	0.685
Protein, g/d	67.2 (9.46)	68.4 (10.2)	70.3 (10.5)	71.9 (10.8)	72.5 (9.95)	**<0.001**
Carbohydrate, g/d	222 (35.4)	223 (33.2)	222 (33.9)	225 (33.3)	226 (29.2)	**0.050**
Total fat, g/d	53.3 (11.6)	53.3 (10.9)	52.1 (10.6)	51.9 (10.3)	51.1 (9.50)	**<0.001**
Saturated fat, g/d	14.7 (3.48)	14.2 (3.02)	13.7 (2.91)	13.4 (2.86)	13.0 (2.63)	**<0.001**
Monounsaturated fat, g/d	20.4 (4.84)	20.2 (4.51)	19.6 (4.37)	19.4 (4.19)	19.0 (3.88)	**<0.001**
Components of a Med score						
Whole grains^e^, g/d	7.76 (11.6)	9.73 (10.4)	11.8 (13.7)	13.4 (10.0)	15.8 (9.26)	**<0.001**
Vegetables (excluded potatoes), g/d	278 (93.9)	313 (97.3)	354 (115)	398 (123)	433 (118)	**<0.001**
Fruits (included juices), g/d	104 (64.2)	124 (76.1)	151 (99.6)	162 (75.3)	192 (75.2)	**<0.001**
Legumes, g/d	27.3 (17.2)	35.9 (23.2)	45.3 (29.8)	48.8 (28.1)	57.7 (27.6)	**<0.001**
Nuts^f^, g/d	1.24 (1.33)	2.05 (2.30)	2.53 (2.46)	3.00 (2.78)	3.59 (2.71)	**<0.001**
Fish, g/d	35.1 (20.8)	42.1 (28.1)	50.0 (37.0)	58.0 (46.3)	63.8 (35.0)	**<0.001**
Monounsaturated to saturated fat ratio	1.40 (0.12)	1.43 (0.14)	1.44 (0.14)	1.45 (0.14)	1.47 (0.13)	**<0.001**
Red and processed meats, g/d	96.4 (34.6)	83.5 (34.3)	77.3 (33.5)	67.4 (29.8)	58.6 (28.2)	**<0.001**
Moderate alcohol drinker, N (%)	0 (0.0)	2 (0.4)	1 (0.2)	3 (0.6)	2 (0.4)	0.255
Women, N	319	333	367	343	316	
Years since menopause, year	9.49 (6.00)	9.89 (5.94)	9.28 (5.56)	9.18 (5.77)	9.81 (6.14)	0.944
Oestrogen user, N (%)	11 (3.4)	21 (6.3)	26 (7.1)	20 (5.8)	21 (6.6)	0.160

We presented continuous variables as Mean (SD) while categorical variables as frequencies (percentage). Linear trends were tested by ANOVA or Chi-square tests as appropriate.

^a^Including 693 men (62.1 ± 5.2, years) and 1,678 women (59.5 ± 4.7, years), 96.8% whom were postmenopausal women.

^b^Smoker were defined as those smoke ≥1 cigarettes daily for at least six consecutive months.

^c^Physical activities included daily activities in occupation, leisure-time, and household-chores was calculated and translated into MET• h/d.

^d^Dietary values presented here were energy-adjusted except for energy intake.

^e^Refers to non-refined cereals, such as graham bread, oats, cereal flakes, etc., calculated as dry weight.

^f^Values was calculated and expressed as proteins.

**Table 2:** Comparisons of covariate-adjusted mean of bone mineral density by quintiles of aMed scores.

	Quintiles of aMed scores					%Diff.^b^	P-Diff	P-trend
	Q1	Q2	Q3	Q4	Q5(highest)			
**aMed Score** (range)	0–2	3	4	5	6–9			
**N (total 2371)**	448	478	502	495	448			
BMD^a^, g/cm^2^								
Whole body								
Model I^c^	1.078 ± 0.005	1.089 ± 0.005	**1.103** ± 0.004^**^	**1.104** ± 0.004^**^	**1.104** ± 0.005^**^	**2.41**	**<0.001**	**<0.001**
Model II^d^	1.081 ± 0.005	1.089 ± 0.004	**1.104** ± 0.004^**^	**1.102** ± 0.004^**^	**1.102** ± 0.005^*^	**1.94**	**<0.001**	**<0.001**
Lumbar Spine L1-4								
Model I^c^	0.859 ± 0.007	0.879 ± 0.007	0.883 ± 0.006	**0.892** ± 0.006^**^	**0.893** ± 0.007^**^	**3.96**	**0.003**	**<0.001**
Model II^d^	0.864 ± 0.006	0.879 ± 0.006	0.886 ± 0.006	0.888 ± 0.006	**0.890** ± 0.006^*^	**3.01**	**0.030**	**0.003**
Total Hip								
Model I^c^	0.817 ± 0.005	0.824 ± 0.005	**0.837** ± 0.005^*^	**0.843** ± 0.005^**^	**0.847** ± 0.005^***^¶	**3.67**	**<0.001**	**<0.001**
Model II^d^	0.821 ± 0.005	0.824 ± 0.005	0.839 ± 0.004	0.839±0.005	**0.844** ± 0.005^**¶^	**2.80**	**0.001**	**<0.001**
Femur neck								
Model I^c^	0.673 ± 0.005	0.684 ± 0.005	0.691 ± 0.005	**0.700** ± 0.005^**^	**0.699** ± 0.005^**^	**3.86**	**<0.001**	**<0.001**
Model II^d^	0.677 ± 0.005	0.685 ± 0.004	0.693 ± 0.004	**0.697** ± 0.004^*^	**0.696** ± 0.005^*^	**2.81**	**0.008**	**<0.001**
Trochanter								
Model I^c^	0.607 ± 0.004	0.613 ± 0.004	0.620 ± 0.004	**0.623** ± 0.004^*^	**0.628** ± 0.004^**^	**3.86**	**0.002**	**<0.001**
Model II^d^	0.610 ± 0.004	0.613 ± 0.004	0.621 ± 0.004	0.621 ± 0.004	**0.626** ± 0.004^*^	**2.62**	**0.018**	**0.001**
Intertrochanter								
Model I^c^	0.978 ± 0.006	0.987 ± 0.006	**1.005** ± 0.006^*^	1.011±0.006^**^	**1.015** ± 0.006^***¶^	**3.78**	**<0.001**	**<0.001**
Model II^d^	0.983 ± 0.006	0.987 ± 0.006	**1.008** ± 0.006^*^	**1.006** ± 0.006^*^	**1.011** ± 0.006^**¶^	**2.85**	**0.001**	**<0.001**
Ward’s triangle								
Model I^c^	0.491 ± 0.006	0.499 ± 0.006	0.509 ± 0.006	**0.516** ± 0.006^*^	0.512 ± 0.006	4.28	**0.016**	**0.002**
Model II^d^	0.495 ± 0.006	0.500 ± 0.006	0.510 ± 0.005	0.513 ± 0.005	0.509 ± 0.006	2.83	0.137	**0.024**

^a^Mean ± SE.

^b^%Diff: percentage difference = (Q5 − Q1)/Q1 × 100%.

^c^Model I: adjusted for age and gender.

^d^Model II: further adjusted for body mass index, martial status, education status, household income, smoking status, calcium supplements use, multivitamin use, physical activities, daily energy intake.*p < 0.05, **p < 0.01, ***p < 0.001, compared with Q1.

^¶^p < 0.05, compared with Q2.

**Table 3:** Comparisons of covariate-adjusted mean of bone mineral density by quintiles of aMed scores stratified by gender.

	quintiles of diet-quality scores					%Diff.^b^	P-Diff.	P-trend	P for interaction
	>Q1	Q2	Q3	Q4	Q5(highest)				
**aMed Score** (range)	0–2	3	4	5	6–9				
**Men**									
**N** = **693**	129	145	135	152	132				
BMD^a^, g/cm^2^									
Whole body	1.161 ± 0.009	1.175 ± 0.008	1.198 ± 0.009	1.180 ± 0.008	1.187 ± 0.009	2.24	**0.042**	**0.037**	0.489
Lumbar Spine L1-4	0.944 ± 0.013	0.955 ± 0.012	0.980 ± 0.012	0.957 ± 0.012	0.974 ± 0.013	3.18	0.227	0.124	0.338
Total Hip	0.892 ± 0.009	0.896 ± 0.009	0.921 ± 0.009	0.915 ± 0.009	0.918 ± 0.009	2.91	0.072	**0.015**	0.725
Femur neck	0.733 ± 0.009	0.742 ± 0.009	0.753 ± 0.009	0.755 ± 0.008	0.753 ± 0.009	2.73	0.344	0.066	0.964
Trochanter	0.658 ± 0.008	0.658 ± 0.008	0.676 ± 0.008	0.671 ± 0.007	0.675 ± 0.008	2.58	0.262	0.065	0.714
Intertrochanter	1.063 ± 0.011	1.070±0.011	1.102 ± 0.011	1.089±0.010	1.091 ± 0.011	2.63	0.079	**0.034**	0.752
Ward’s triangle	0.510 ± 0.011	0.510 ± 0.011	0.525 ± 0.011	0.526 ± 0.010	0.518 ± 0.011	1.59	0.723	0.382	0.968
**Women**									
**N** = **1678**	319	333	367	343	316				
BMD^a^, g/cm^2^									
Whole body	1.048 ± 0.005	1.055 ± 0.005	1.065 ± 0.005	1.069 ± 0.005	1.067 ± 0.005	1.81	**0.023**	**0.002**	
Lumbar Spine L1-4	0.832 ± 0.007	0.850 ± 0.007	0.846 ± 0.007	0.859 ± 0.007	0.856 ± 0.007	2.88	0.069	**0.012**	
Total Hip	0.793 ± 0.005	0.795 ± 0.005	0.805 ± 0.005	0.807 ± 0.005	0.813 ± 0.005	2.40	0.056	**0.003**	
Femur neck	0.656 ± 0.005	0.662 ± 0.005	0.667 ± 0.005	0.672 ± 0.005	0.673 ± 0.005	2.59	0.090	**0.006**	
Trochanter	0.591 ± 0.004	0.595 ± 0.004	0.598 ± 0.004	0.599 ± 0.004	0.607 ± 0.004	2.71	0.136	**0.010**	
Intertrochanter	0.952 ± 0.007	0.954 ± 0.007	0.969 ± 0.006	0.971 ± 0.007	0.978 ± 0.007	2.73	**0.026**	**0.001**	
Ward’s triangle	0.491 ± 0.007	0.496 ± 0.006	0.503 ± 0.006	0.506 ± 0.006	0.506 ± 0.007	3.05	0.379	0.052	

All analyses were adjusted for age, body mass index, marital status, education status, household income, smoking status, calcium supplement use, multivitamin use, physical activity, and daily energy intake. For women, years since menopause and oral estrogen use were further adjusted.

^a^Mean ± SE.

^b^%Diff.: percentage difference = (Q5 − Q1)/Q1 × 100%.

**Table 4:** Comparisons of covariate-adjusted mean of bone mineral density by different aMed models (N = 2371).

	Whole body	Lumbar spine	Total hip	Femur neck	Trochanter	Intertrochanter	Ward’s triangle
BMD, g/cm^2^^a^							
*aMed score, Model I*							
Quintiles 1^a^	1.081 ± 0.005	0.864 ± 0.006	0.821 ± 0.005	0.677 ± 0.005	0.610 ± 0.004	0.983 ± 0.006	0.495 ± 0.006
Quintiles 5 (highest) Diff.^b^	0.021 ± 0.005	0.026 ± 0.006	0.023 ± 0.005	0.024 ± 0.005	0.016 ± 0.004	0.028 ± 0.006	0.014 ± 0.006
%Diff. I^c^	1.94^*^	3.01^*^	2.80^**^	2.81^*^	2.62^**^	2.85^**^	2.83
*aMed score, Model II*							
Quintiles 1^a^	1.083 ± 0.004	0.870 ± 0.006	0.822 ± 0.004	0.680 ± 0.004	0.611 ± 0.003	0.985 ± 0.005	0.497 ± 0.004
Quintiles 5 (highest) Diff.^b^	0.018 ± 0.004	0.021 ± 0.005	0.022 ± 0.004	0.018±0.004	0.016 ± 0.003	0.026 ± 0.004	0.017 ± 0.004
%Diff. II^c^	1.66^**^	2.41^**^	2.67^**^	2.65^**^	2.62^**^	2.64^**^	3.42
*aMed score, Model III*							
Quintiles 1^a^	1.082 ± 0.004	0.866 ± 0.005	0.822 ± 0.004	0.677 ± 0.004	0.610 ± 0.003	0.984 ± 0.005	0.495 ± 0.005
Quintiles 5 (highest) Diff.^b^	0.023 ± 0.004	0.025±0.005	0.024 ± 0.004	0.022 ± 0.004	0.017 ± 0.003	0.029 ± 0.003	0.018 ± 0.003
%Diff. III^c^	2.13^***^	2.89^**^	2.92^***^	3.25^***^	2.79^**^	2.95^**^	3.64
*aMed score, Model IV*							
Quintiles 1^a^	1.081 ± 0.004	0.866 ± 0.005	0.820 ± 0.004	0.677 ± 0.004	0.610 ± 0.003	0.981 ± 0.005	0.494 ± 0.005
Quintiles 5 (highest) Diff.^b^	0.020 ± 0.004	0.025 ± 0.006	0.024 ± 0.004	0.021 ± 0.004	0.015 ± 0.003	0.031 ± 0.005	0.016 ± 0.005
%Diff. IV^c^	1.85^**^	2.89^**^	2.93^***^	3.10^**^	2.46^*^	3.16^***^	3.24
*aMed score, Model V*							
Quintiles 1^a^	1.082 ± 0.004	0.869±0.005	0.821 ± 0.004	0.680 ± 0.004	0.611 ± 0.003	0.982 ± 0.005	0.495 ± 0.005
Quintiles 5 (highest) Diff.^b^	0.022 ± 0.004	0.020 ± 0.006	0.020 ± 0.004	0.017 ± 0.004	0.013 ± 0.004	0.026 ± 0.005	0.015 ± 0.005
%Diff. V^c^	2.03^**^	2.30	2.44^**^	2.50^*^	2.13	2.65^**^	3.03
*aMed score, Model VI*							
Quintiles 1^a^	1.080±0.005	0.864 ± 0.006	0.821 ± 0.005	0.677 ± 0.005	0.610 ± 0.004	0.983 ± 0.006	0.495 ± 0.005
Quintiles 5 (highest) Diff.^b^	0.022 ± 0.004	0.027 ± 0.006	0.023 ± 0.004	0.019 ± 0.004	0.017 ± 0.004	0.028 ± 0.005	0.014±0.005
%Diff. VI^c^	2.03^**^	3.11^*^	2.80^**^	2.79^*^	2.78^*^	2.85^**^	2.83
*aMed score, Model VII*							
Quintiles 1^a^	1.076 ± 0.007	0.857 ± 0.009	0.820 ± 0.007	0.680 ± 0.006	0.610 ± 0.006	0.980 ± 0.008	0.495 ± 0.008
Quintiles 5 (highest) Diff.^b^	0.046±0.007	0.056 ± 0.009	0.039 ± 0.007	0.037 ± 0.006	0.027 ± 0.006	0.051 ± 0.008	0.035 ± 0.008
%Diff. VII^c^	4.28^***^	6.53^***^	4.76^***^	5.44^***^	4.43^**^	5.20^***^	7.07^*^
%Diff. increment^d^	**121**	**117**	**70.0**	**93.6**	**69.1**	**82.5**	**150**

All analyses were adjusted for age, body mass index, marital status, education status, household income, smoking status, calcium supplement use, multivitamin use, physical activity, and daily energy intake. In Model I, aMed scores were constructed by the original 9 components (whole grain, vegetables excluded potato, fruits include juices, legumes, nuts, fish, monounsaturated to saturated fat ratio, red and processed meat, and moderate alcohol consume). Vegetable, legumes, fish, monounsaturated to saturated ratio, moderate alcohol consume was each excluded from Model I in Model II, III, IV, V, VI, respectively, and excluded together in the Model VII. Score ranges from 0–9, 0–8, 0–8, 0–8, 0–8, 0–8, and 0–4, respectively in Model I–VII.

^a^Mean ± SE.

^b^Mean difference (Q5–Q1) ± SE.

^c^%Diff.: percentage difference = (Q5-Q1)/Q1 × 100%.

^d^%Diff. increment = ([%Diff.VII–%Diff.I])/%Diff.I) × 100%. *p < 0.05, **p < 0.01, ***p < 0.001, compared with Q1.
