# Supplementary material for: Adherence to the Mediterranean diet is associated with a higher BMD in middle-aged and elderly Chinese
Source: Sci Rep. 2016 May 9;6:25662. doi: 10.1038/srep25662 (PMC4860711; doi:10.1038/srep25662)
Supplement: Supplemental Table 1 [file srep25662-s1.doc]

**Adherence to the Mediterranean diet is associated with a higher BMD in middle-aged and elderly Chinese**

Geng-dong Chen, Xiao-wei Dong, Ying-Ying Zhu, Hui-yuan Tian, Juan He, Yu-ming Chen.

**Supplemental Table 1. Comparisons of covariate-adjusted mean of bone mineral density by individual aMed component (N=2371).**

|  | Whole body | Lumbar spine | Total hip | Femur neck | Trochanter | Intertrochanter | Ward’s triangle |
| --- | --- | --- | --- | --- | --- | --- | --- |
| BMD ^a^, g/cm^2 a^ |  |  |  |  |  |  |  |
| Whole grains score |  |  |  |  |  |  |  |
| 0 (N=1185) | 1.091±0.003 | 0.878±0.004 | 0.830±0.003 | 0.687±0.003 | 0.617±0.002 | 0.994±0.004 | 0.503±0.003 |
| 1 (N=1186) ^b^ | 1.101±0.003 | 0.885±0.004 | 0.837±0.003 | 0.693±0.003 | 0.620±0.002 | 1.004±0.004 | 0.508±0.003 |
| %Diff. ^c^ | **0.92^*^** | 0.91 | 0.84 | 0.87 | 0.49 | 1.01 | 0.99 |
| Vegetables score |  |  |  |  |  |  |  |
| 0 (N=1185) | 1.094±0.003 | 0.879±0.004 | 0.832±0.003 | 0.689±0.003 | 0.617±0.002 | 0.996±0.003 | 0.509±0.003 |
| 1 (N=1186) ^b^ | 1.098±0.003 | 0.884±0.004 | 0.836±0.003 | 0.691±0.003 | 0.620±0.002 | 1.003±0.003 | 0.502±0.003 |
| %Diff. ^c^ | 0.37 | 0.59 | 0.48 | 0.29 | 0.49 | 0.70 | -1.38 |
| Fruits score |  |  |  |  |  |  |  |
| 0 (N=1185) | 1.087±0.003 | 0.874±0.004 | 0.827±0.003 | 0.683±0.003 | 0.615±0.002 | 0.990±0.003 | 0.498±0.003 |
| 1 (N=1186) ^b^ | 1.104±0.003 | 0.889±0.004 | 0.840±0.003 | 0.696±0.003 | 0.622±0.002 | 1.008±0.003 | 0.513±0.003 |
| %Diff. ^c^ | **1.56^**^****^*^** | **1.83^**^** | **1.57^**^** | **1.90^**^** | 1.14 | **1.82^**^** | **3.01^**^** |
| Legume score |  |  |  |  |  |  |  |
| 0 (N=1185) | 1.100±0.003 | 0.885±0.004 | 0.837±0.003 | 0.694±0.003 | 0.621±0.002 | 1.002±0.004 | 0.510±0.004 |
| 1 (N=1186) ^b^ | 1.092±0.003 | 0.878±0.004 | 0.831±0.003 | 0.686±0.003 | 0.616±0.002 | 0.996±0.004 | 0.501±0.004 |
| %Diff. ^c^ | -0.72 | -0.79 | -0.72 | -1.15 | -0.81 | -0.60 | -1.76 |
| Nut score |  |  |  |  |  |  |  |
| 0 (N=1185) | 1.090±0.003 | 0.875±0.004 | 0.826±0.003 | 0.683±0.003 | 0.613±0.002 | 0.989±0.004 | 0.499±0.004 |
| 1 (N=1186) ^b^ | 1.102±0.003 | 0.888±0.004 | 0.842±0.003 | 0.697±0.003 | 0.624±0.002 | 1.009±0.004 | 0.511±0.004 |
| %Diff. ^c^ | **1.10^**^** | **1.49^*^** | **1.94^***^** | **2.05^**^** | **1.79^**^** | **2.02^***^** | **2.40^**^** |
| Fish score |  |  |  |  |  |  |  |
| 0 (N=1185) | 1.092±0.003 | 0.880±0.004 | 0.833±0.003 | 0.689±0.003 | 0.616±0.002 | 0.999±0.003 | 0.504±0.003 |
| 1 (N=1186) ^b^ | 1.100±0.003 | 0.883±0.004 | 0.835±0.003 | 0.690±0.003 | 0.621±0.002 | 1.000±0.003 | 0.506±0.003 |
| %Diff. ^c^ | 0.73 | 0.34 | 0.24 | 0.15 | 0.81 | 0.10 | 0.40 |
| Monounsaturated to saturated fat ratio score |  |  |  |  |  |  |  |
| 0 (N=1185) | 1.095±0.003 | 0.880±0.004 | 0.831±0.003 | 0.687±0.003 | 0.616±0.002 | 0.996±0.003 | 0.503±0.003 |
| 1 (N=1186) ^b^ | 1.096±0.003 | 0.883±0.004 | 0.837±0.003 | 0.693±0.003 | 0.621±0.002 | 1.002±0.003 | 0.508±0.003 |
| %Diff. ^c^ | 0.00 | 0.34 | 0.72 | 0.87 | 0.81 | 0.60 | 0.99 |
| Red and processed meats score |  |  |  |  |  |  |  |
| 0 (N=1185) | 1.090±0.003 | 0.872±0.004 | 0.828±0.003 | 0.684±0.003 | 0.616±0.002 | 0.993±0.003 | 0.499±0.004 |
| 1 (N=1186) ^b^ | 1.101±0.003 | 0.891±0.004 | 0.839±0.003 | 0.696±0.003 | 0.623±0.002 | 1.005±0.003 | 0.512±0.004 |
| %Diff. ^c^ | **1.01^**^** | **2.18^**^** | **1.33****^**^** | **1.75^**^** | **1.14^*^** | **1.21^*^** | **2.61^**^** |
| Moderate alcohol consume score |  |  |  |  |  |  |  |
| 0 (N=2363) | 1.096±0.002 | 0.882±0.003 | 0.834±0.002 | 0.690±0.002 | 0.618±0.002 | 0.999±0.003 | 0.506±0.003 |
| 1 (N=8) ^b^ | 1.041±0.035 | 0.815±0.049 | 0.824±0.036 | 0.698±0.034 | 0.616±0.030 | 0.982±0.044 | 0.486±0.043 |
| %Diff. ^b^ | -5.02 | -7.60 | -1.20 | 1.16 | 1.29 | -0.32 | -3.95 |

All analyses were adjusted for age, sex, body mass index, marital status, education status, household income, smoking status, calcium supplement use, multivitamin use, physical activity, and daily energy intake.

^a^ *Mean ±SE.*

^b^ *Mean difference* (Score 1 – Score 0) *± SE.*

^c^ %Diff: percentage difference = (Score 1 – Score 0)/Score 0×100%.

*: p<0.05, **: p<0.01, ***: p<0.001, significance of mean difference compared with Q1;
